# β-nicotinamide mononucleotide rescues the quality of aged oocyte and improves subsequent embryo development in pigs

**DOI:** 10.1371/journal.pone.0291640

**Published:** 2023-10-05

**Authors:** Leyi Li, Qinghe Han, Yurong Chen, Meng Zhang, Luyao Wang, Xinglan An, Sheng Zhang, Yanhui Zhai, Xiangpeng Dai, Bo Tang, Ziyi Li, Guanghong Xie

**Affiliations:** 1 Key Laboratory of Zoonosis Research, Ministry of Education, College of Veterinary Medicine, Jilin University, Changchun, 130021, Jilin, China; 2 Radiology Department, The second hospital of Jilin University, Changchun, 130041, P. R. China; 3 Key Laboratory of Organ Regeneration and Transplantation of Ministry of Education, First Hospital, Jilin University, Changchun, 130021, Jilin, China; China Agricultural University, CHINA

## Abstract

Oocyte senescence alters the shape and function, thereby weakening the fertilization potential. Nicotinamide mononucleotide (NMN) reverses age-related dysfunctions in various organs. Studies had shown long-term administration of NMN reduced the physiological decline associated in aged mice and reversed the aging of the ovaries. However, the protective effect of NMN on aged porcine oocytes is still unclear. In this study, we investigated the effects of NMN on aging porcine oocytes and subsequent embryonic development. We established a model of senescence of porcine oocytes after ovulation by extending the culture time in vitro. NMN supplementation significantly reduced reactive oxygen species (ROS) levels in senescence oocytes and increased the mRNA levels of antioxidant genes *SOD1* and *Cat*. The mitochondrial membrane potential of aged oocytes treated with NMN was increased compared with that of untreated oocytes. In addition, the mRNA level of apoptosis-related gene Bax was significantly decreased in senescence oocytes treated with NMN, while the mRNA level of anti-apoptosis-related gene BCL-2 was significantly increased. Furthermore, NMN supplementation enhanced the subsequent development ability of senescent oocytes during in vitro aging. Compared with untreated senescent oocytes, the blastocyst formation rate and pluripotent genes of senescent oocytes treated with NMN were significantly increased. Taken together, these results suggest that NMN is beneficial for delaying the aging process in porcine oocytes.

## 1 Introduction

During the process of meiosis, mammalian oocytes undergo two key events in the meiosis stage I, namely the nuclear envelope breakdown and the extrusion of the first polar body, and enter the second stage of meiosis to wait for fertilization [1,2]. If the oocytes are not fertilized in time, the discharged oocytes will undergo a time-dependent degradation process in vivo or in vitro, which is called oocyte senescence [3]. Oocyte senescence leads to a decrease in the content of NAD^+^, resulting in a decrease in the activity of maturation promoting factors, abnormal cell membrane skeleton, ROS production, mitochondrial dysfunction, epigenetic modification changes [4–7]. These changes will eventually bring about oocyte spindle/chromosome abnormalities, weakened fertilization ability, and abnormal embryo and fetal development [8–12]. Studies have shown that the addition of certain antioxidants, such as L-cysteine, resveratrol or melatonin, can increase the expression level of NAD^+^ in oocytes and promote subsequent development ability [6,13–18].

NMN is a nucleotide with a molecular weight of 334.221g/mol, which is formed by the reaction of a phosphate group with ribose-containing nucleosides and nicotinamide under natural conditions [19]. NMN is a key intermediate of NAD^+^, which can reverse the mitochondrial homeostasis, ROS production, DNA repair caused by insufficient NAD^+^ and cell survival defects [20]. It was found that NMN significantly improved the morphological defects of oocytes and progeny survival rate in aged mice [21,22]. However, the effect of NMN on the developmental capacity of porcine aging oocytes and subsequent embryos has not been determined.

In the present study, we evaluated that whether NMN prevents porcine oocytes from in vitro ageing. Firstly, mature oocytes were cultured in vitro for 24 h and 48 h to construct aged porcine oocytes. Different concentrations of NMN were used to detect the senescence rate of oocytes, and the optimal concentration of NMN was selected for the follow-up study. In addition, the cytoskeletal integrity, spindle formation and DNA arrangement, ROS level and mitochondrial function of porcine oocytes supplemented with NMN were detected by immunofluorescence staining. Finally, the effect of NMN on early embryonic development of aged porcine oocytes was studied by parthenogenetic activation. This study provides a theoretical basis for the quality control of clinical assisted reproductive oocytes.

## 2 Material and methods

### Chemicals and reagents

All chemicals and culture media were purchased from Sigma Chemical Company (St. Louis, MO) unless stated otherwise.

### Collection and in vitro maturation of porcine oocytes

The porcine ovaries were collected from the local slaughterhouse. porcine ovaries were stored in a thermos flask transported to the laboratory within 1–2 h. The 10ml syringe was used to extract cumulus cell complexes (COCs) from the follicles, and COCs with three layers of cumulus cells were placed in a 37° C oven for precipitation for 10-15min, then washed twice in pBS containing 10% fetal bovine serum. After that, the oocytes were cultured in vitro (medium I, with hormones) in the incubator at 38.5° C, 5% CO_2_ and 95% air for 22–24 h, and then transferred to medium II (without hormones) for in vitro maturation culture until 42–44 h. The mature medium I was composed of TCM-199 supplemented with 0.9 mM sodium pyruvate, 26 mM sodium bicarbonate, 3.1 mM glucose, 10 μg/ml epidermal growth factor, 50 μg/ml luteinizing hormone, 50 μg/ml follicle-stimulating hormone, 0.03% bovine serum albumin (BSA), and 0.1% penicillin/streptomycin (Gibco) and polyvinyl alcohol (PVA), while the mature medium II was the same with mature medium I without hormones.

### In vitro aged and NMN treatment

The cumulus cells of matured oocytes were removed by the PBS supplemented with 0.15% (w/v) hyaluronidase and 0.15% (w/v) PVA for 2 min, only oocytes with first polar bodies were used for subsequent studies. The matured oocytes were cultured in porcine Zygote Medium (pZM-3) [23] with or without NMN (S5259, Selleck, USA) (1μmol/L, 10μmol/L, 100μmol/L) for an additional 24 h or 48 h at 38.5° C with 5% CO_2_. The aged ratio of oocytes was detected at 24 h and 48 h.

### Parthenogenetic activation and in vitro culture of porcine oocytes

parthenogenetic activation was performed on fresh MII oocytes and aged porcine oocytes (0.3 M mannitol, 1 mM CaCl_2_ 2H_2_O, 1 mM MgCl_2_ 6H_2_O and 0.5 mM HEPES). The porcine oocytes were arranged in the electrophoresis tank in sequence, and two continuous DC pulses of 1.2 kV/cm were activated by the ECM2001 electrofusion instrument (ECM2001, BTX, USA) for 30 μs. During electrical activation, the orientation of oocyte and polar body was not perpendicular to electrode. After activation, the embryos were washed subsequently, the embryos were placed in embryo culture medium pZM-3, cultured at 38.5° C, 5% CO_2_ for 7 days, and developed to the blastocyst stage.

### Extraction of RNA and quantitative PCR

Total RNA was extracted from 180–200 zona pellucida-removed porcine oocytes using RNeasy Mini kit (Qiagen, Hilden, Germany), and cDNA was synthesized with TransScript All-in-One-First-Strand cDNA (TranGen Biotech, Beijing, China), each 20 μl reaction system contains 6 μl total RNA, 4 μl TransScript All-in-One SuperMix for qPCR, 1 μl gDNA remover, and 9 μl RNase-free Water. The cDNA amplification system was: 45° C for 15 min and 85° C for 15 s. QPCR was performed on 96-well plates. Each 20 μl reaction system contained 10 μl SYBR green premix, 2 μl cDNA 2 μl primers, and 6 μl non-enzymatic water. The amplification conditions were as follows: denaturation at 50° C at 120s, 95° C at 120s, pCR at 40 cycles (95° C at 15 s and 60° C at 60s), melt-curve 95° C at 15 s, 60° C at 60 s and 95° C at 95 s, then maintained at 4° C. The relative expression of each gene was calculated using the 2^-ΔΔCT^ method.

### Detection of ROS

To determine the level of intracellular ROS, fresh or aged oocytes were incubated in pBS for 30 minutes with 10 μM Reactive Oxygen Species Assay Kit (Beyotime Biotechnology, Shanghai, China). The oocytes were washed 3 times in PBS containing 0.1% PVP. Fluorescence detection was performed by a fluorescence microscope (Nikon, Tokyo, Japan), followed by spectroscopic analysis (green fluorescence, UV filters, 490 nm). The fluorescence intensity of each oocyte was measured under the same scan settings. Image J software was used to analyse the fluorescence intensity of oocytes, and the mean value was calculated after separating the channels.

### Detection of mitochondrial content

For mitochondrial content staining, the oocytes were immersed in MitoTracker Red CMXRos (Thermo Fisher Scientific, Waltham, MA, USA) in a dark environment for 30 minutes at 38.5°C and 5% CO_2_, and 0.1% PVP in PBS, then the oocytes were washed 3 times with PBS containing 0.1% PVP for 2–3 minutes each time. The mitochondrial content detection was performed by a fluorescence microscope (Nikon, Tokyo, Japan). The fluorescence intensity of each oocyte was measured under the same scan settings. Image J software was used to analyse the fluorescence intensity of oocytes, and the mean value was calculated after separating the channels.

### Detection of mitochondrial membrane potential

The oocytes were stained using JC-1 staining kit (Beyotime Biotechnology, Shanghai, China) according to the manufacturer’s instructions. The 1 μl of dye was diluted with 180 μl of ultrapure water, and 40 μl of JC-1 staining buffer was added to make a working solution. Fresh or aged oocytes were washed twice with PBS and then stained with JC-1. The detection of mitochondrial membrane potential was performed by a fluorescence microscope (Nikon, Tokyo, Japan). The fluorescence intensity of each oocyte was measured under the same scan settings. Image J software was used to analyse the fluorescence intensity of oocytes, and the mean value was calculated after separating the channels.

### Analysis of immunofluorescence and confocal laser scanning microscopy

porcine oocytes removed from zona pellucida were fixed in 4% paraformaldehyde for 30 minutes, and infiltrated with 1% Triton X-100/PBS (v/v) for 20 minutes. The oocytes were blocked with PBS containing 1% bovine serum albumin (BSA) at 37°C for 1 h-1.5 h. The oocytes were treated with anti-a-tubulin-FITC antibody (Sigma, F2168, 1:200) and anti-phalloidin-TRITC antibody (Beyotime, C1033, 1:200) for overnight. The oocytes were incubated with the secondary antibody at 37°C for 1.5 h, and was counter-stained with propidium iodide (10 μg/ml) at room temperature for 10 minutes. The primary and secondary antibodies were diluted with 0.1% BSA in PBS. Fluorescence was detected Chromosomal arrangement is performed by a fluorescence microscope (Nikon, Tokyo, Japan) or a confocal microscope (LSM 8800, Zeiss, Oberkochen, Germany). The fluorescence intensity of each oocyte was measured under the same scan settings. Image J software was used to analyse the fluorescence intensity of oocytes, and the mean value was calculated after separating the channels.

### Statistical analysis

Data are presented as the mean ± standard deviations (SD), unless otherwise indicated. One-way ANOVA was used to compare the statistical differences between experimental groups. All the experimental materials were oocytes obtained from the ovaries of different pigs. All experiments were repeated at least three times, p < 0.05 was considered statistically significant; p < 0.01 was considered extremely significant.

## 3 Results

### 3.1 NMN rescued porcine oocyte senescence

**Fig 1:**
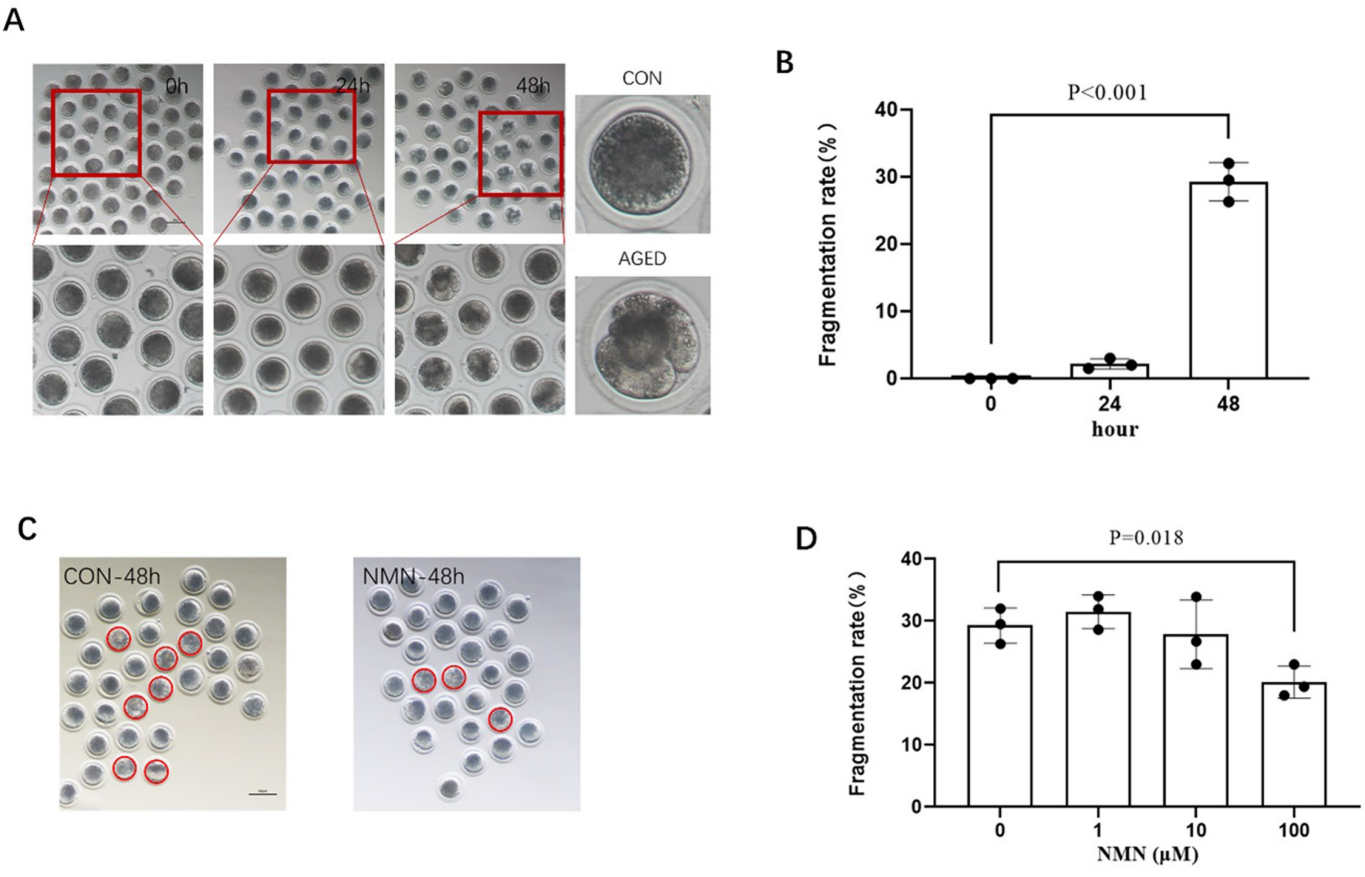
NMN rescued aged-induced fragmentation of porcine oocytes. (A) Oocyte morphologies and fragmentation rate (B) in aged 0 h, 24 h, and 48 h groups; (C) Oocyte morphologies at CON-48 h and 100 μm NMN groups. The red circles represent aging oocytes; (D) The fragmentation rate of aged 48 h oocytes treated with different concentrations of NMN (0, 1, 10 or 100 μm). The red dotted circle indicated fragmented oocytes. Scale bars represent 100 μm. *p < 0.05, **p < 0.01.

After the oocytes were cultured in vitro for 42–44 h, mature oocytes with first polar body were selected and placed in PZM-3 for 24 h and 48 h, respectively. As shown in Fig 1A and 1B, cleaved and fragmented oocytes were regarded as aged oocyte. The proportion of aged oocytes was markedly higher than that of fresh oocytes. With the extension of in vitro culture time, the aged ratio of oocytes gradually increased (0.00% control vs. 2.17±0.76% 24 h aged vs. 29.27±2.86% 48 h aged; p<0.001; Fig 1A and 1B; n = 3). Subsequently, senescent oocytes were treated with NMN concentrations of 1 μmol/L, 10 μmol/L and 100 μmol/L, respectively. The results showed that 100 μmol/L NMN rescued the aged ratio of porcine oocytes at 48 h (29.27±2.86% 48 h aged vs. 20.13±2.58% 48 h aged+100 μmol/L NMN; p<0.05; Fig 1C and 1D; n = 3), indicating that NMN rescued oocyte senescence from fragmentations.

### 3.2 NMN reduced the level of ROS in aged porcine oocytes

**Fig 2:**
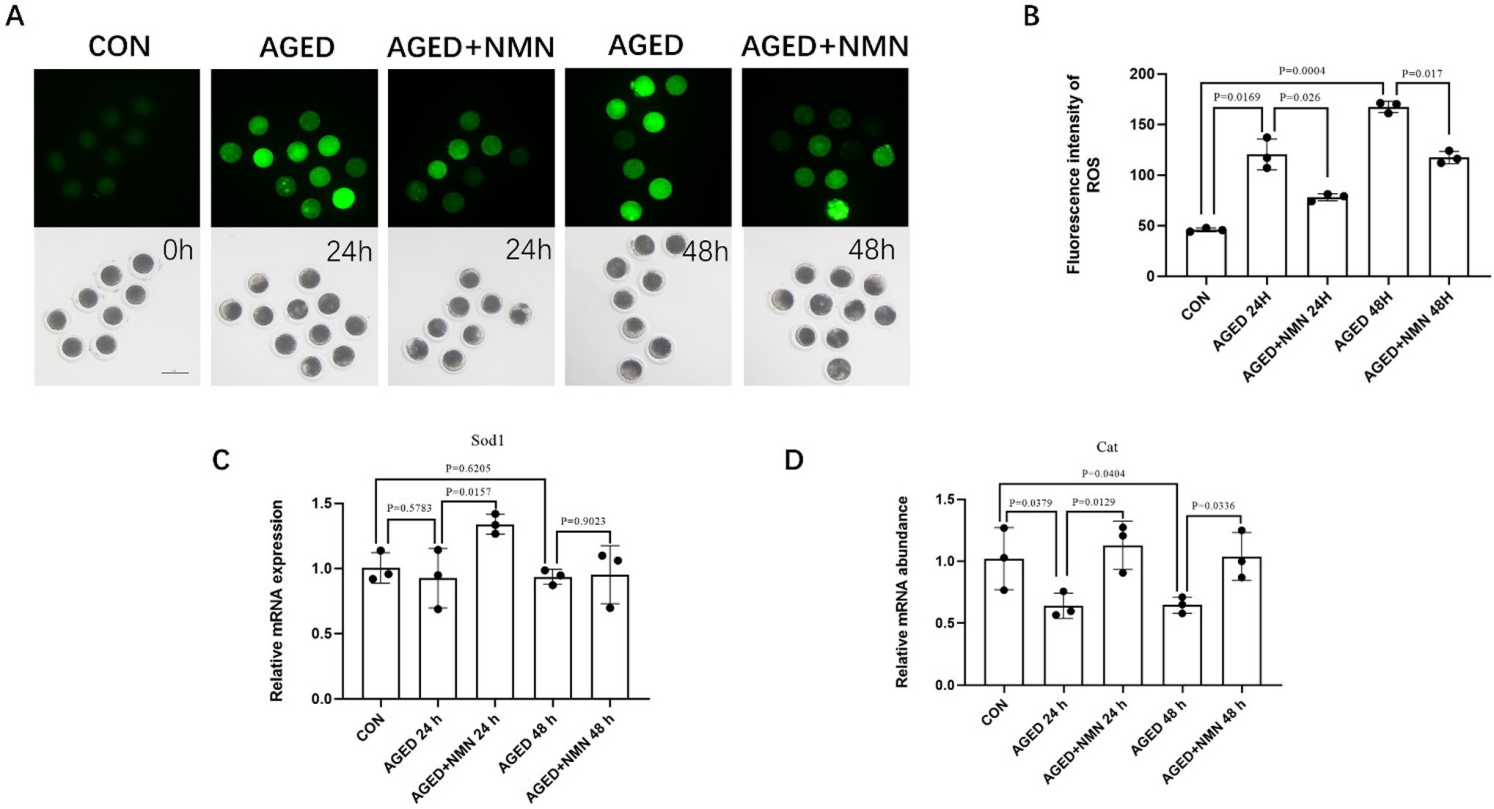
NMN reduced the level of ROS in aged porcine oocytes. Images (A) and ROS level (B) at 0 h, AGED-24 h, AGED+NMN-24 h, AGED-48 h and AGED+NMN-48 h oocytes. Scale bars represented 100 μm. The expression of anti‐oxidative stress genes *SOD1* (C) and *Cat* (D) in fresh and aged oocytes. *p < 0.05, **p < 0.01 indicated significant differences.

Increased levels of ROS damaged the quality of oocytes. Therefore, we used H_2_DCF-DA to detect the expression level of ROS in porcine oocytes. As shown in Fig 2A and 2B, with the prolongation of aged time, the level of ROS in porcine oocytes gradually increased when compared with the control group (45.80±1.81 control vs. 120.3±15.28 24 h aged vs. 167.3±5.51 48 h aged; Fig 2B; p<0.01; n = 3). The expression of ROS in the NMN-treated group showed a decreasing trend at 24 h or 48 h (120.3±15.28 24 h aged vs. 78.00±3.61 24 h aged+NMN; 167.3±5.51 48 h aged vs. 117.0±6.11 48 h aged+NMN; Fig 2B; p<0.01; n = 3). Meanwhile, it was found that NMN increased the expression of anti-oxidant genes *SOD1* (0.93±0.23 24 h aged vs. 1.34±0.08 24 h aged+NMN; Fig 2C; p<0.05; n = 3) and *Cat* (0.64±0.10 24 h aged vs. 1.128±0.19 24 h aged+NMN; 0.65±0.07 48 h aged vs. 1.04±0.20 48 h aged+NMN; Fig 2D; p<0.05; n = 3). These results indicated that NMN supplementation can reduce ROS levels and enhance the expression of antioxidant genes in aged porcine oocytes.

### 3.3 NMN enhanced mitochondrial function of aged porcine oocytes

**Fig 3:**
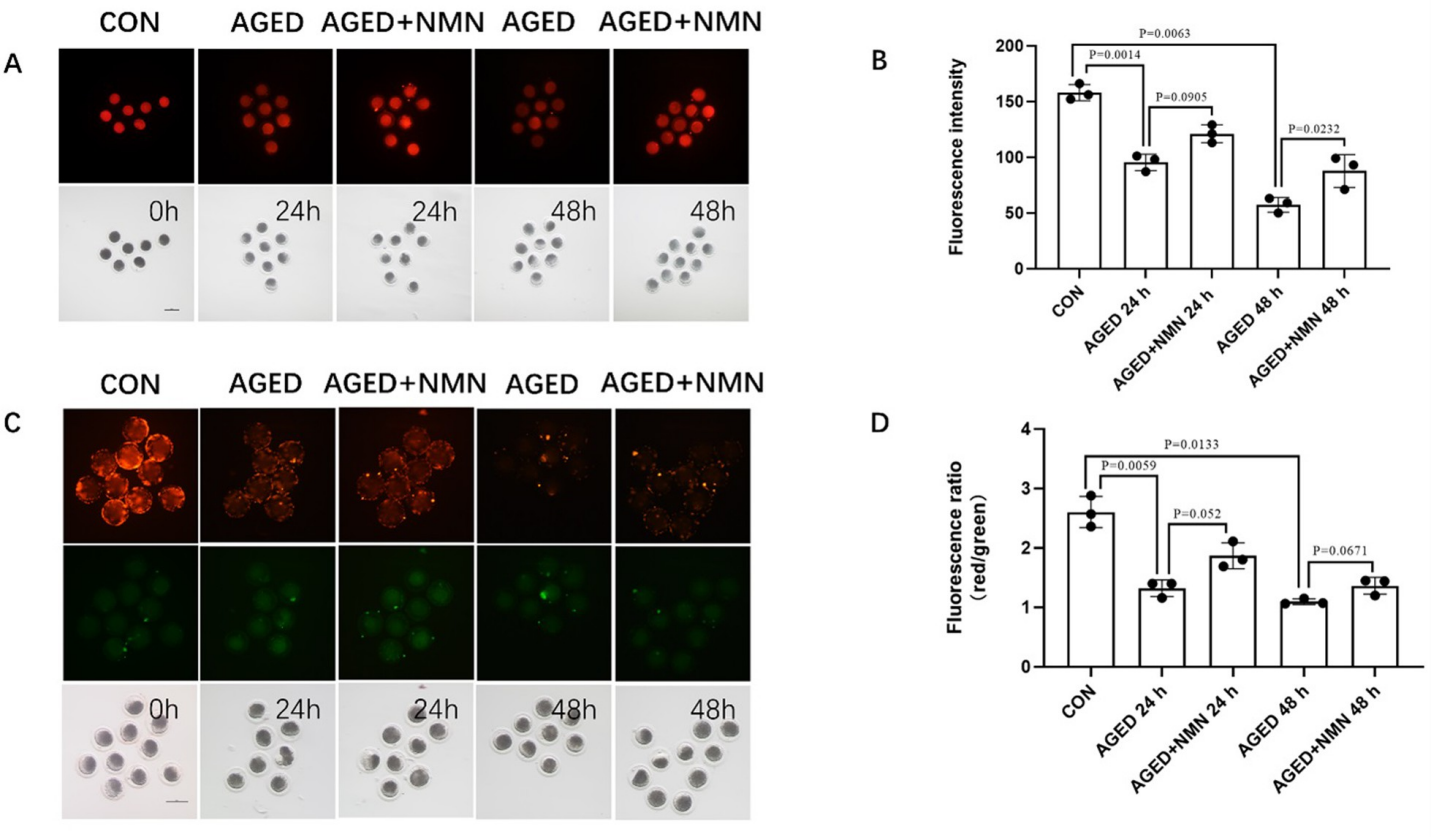
NMN rescued porcine oocyte mitochondrial dysfunction caused by aged. Images (A) of mitochondrial content level (B) at 0 h, AGED-24 h, AGED+NMN-24 h, AGED-48 h and AGED+NMN-48 h oocytes. Scale bars represented 100 μm. Images (C) of mitochondrial membrane potential level (D) at 0 h, AGED-24 h, AGED+NMN-24 h, AGED-48 h and AGED+NMN-48 h oocytes. *p < 0.05 indicated significant differences. Scale bars represented 100 μm.

Mitochondria are the main place for ATp production and are essential for the development of oocytes. Therefore, we used Mito Tracker Red CMXRos and JC-1 to detect mitochondrial activity and mitochondrial membrane potential. With the extension of aging time, the mitochondrial content (158.0±7.21 control vs. 95.33±7.37 24 h aged vs. 57.33±6.66 48 h aged; Fig 3B; p<0.05; n = 3) and the fluorescence intensity of the mitochondrial membrane potential (2.60±0.26 control vs. 1.320±0.14 24 h aged vs. 1.01±0.05 48 h aged; Fig 3D; p<0.05; n = 3) in porcine oocytes gradually decreased when compared with the control group. Compared with the untreated group, the mitochondrial content (95.33±7.37 24 h aged vs. 121.0±8.00 24 h aged+NMN; p = 0.091; 57.33±6.66 48 h aged vs. 87.67±14.74 48 h aged+NMN; Fig 3B; p<0.05; n = 3) increased significantly in the NMN-treated group at 48 h. Although NMN has no significant effect on mitochondrial membrane potential of aged oocytes, it still has some improvement effect. These results indicated that NMN have a protective effect on oocyte mitochondria during the aged process.

### 3.4 NMN reduced cytoskeletal damage of aged porcine oocytes

**Fig 4:**
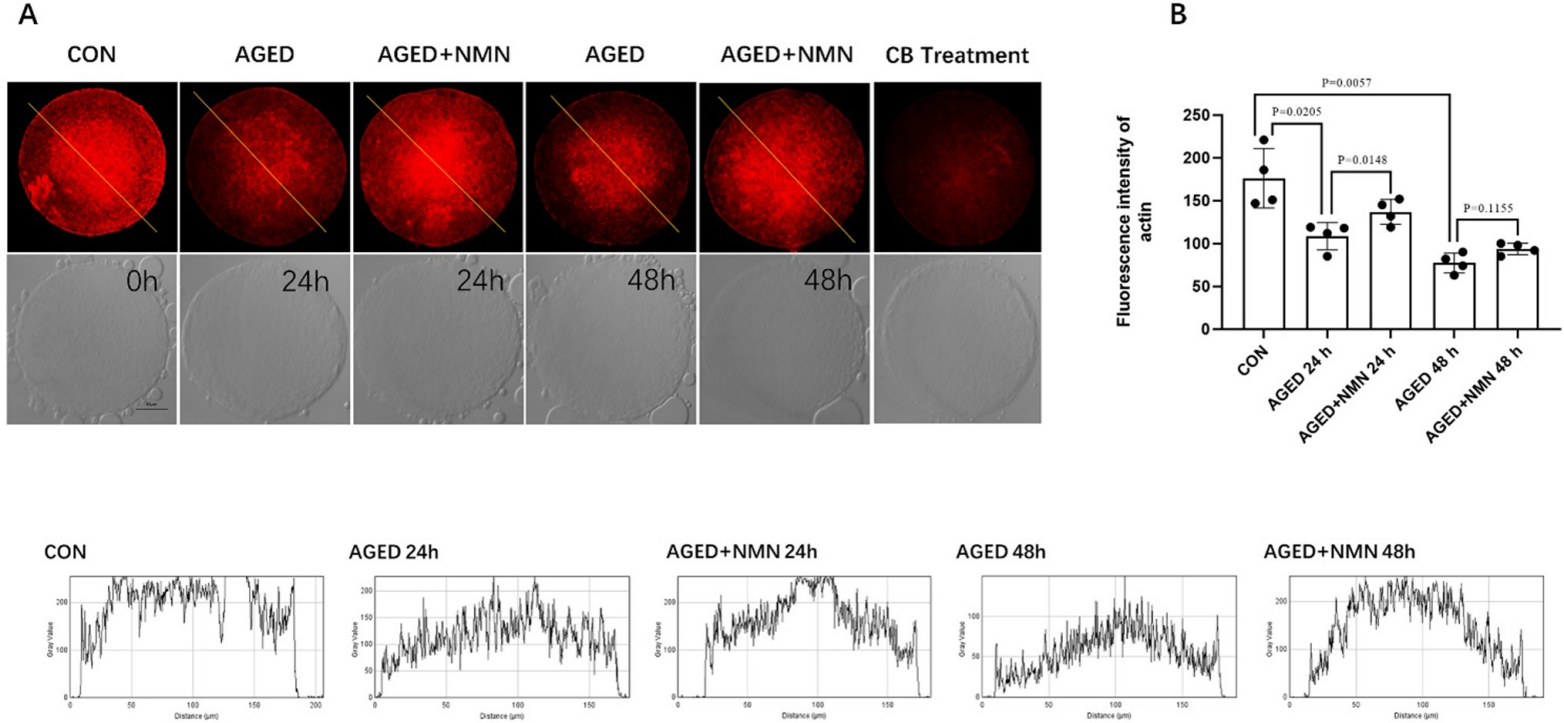
NMN rescued the cytoskeletal damage of aged porcine oocytes. Representative confocal images (A) and fluorescence intensity (B) of actin at 0 h, AGED-24 h, AGED+NMN-24 h, AGED-48 h and AGED+NMN-48 h oocytes. *p < 0.05 indicated significant differences. Scale bars represented 50 μm.

In normal oocytes, the actin cytoskeleton plays an important role in asymmetric spindle positioning and cortical polarization. In order to test whether NMN delays the damage of the cell membrane skeleton of aged porcine oocytes, phalloidin-TRITC was used to detect the damage of the cell membrane skeleton of porcine oocytes. As shown in Fig 4A and 4B, the actin signal of oocytes decreased at 48 h (176.3±34.60 control vs. 108.5±15.97 24 h aged vs. 77.25±11.53 48 h aged; Fig 4B; p<0.05; n = 3). However, compared with the untreated group, NMN rescue actin at 24 h (Fig 4A). Statistical analysis showed that actin intensity of aged porcine oocytes decreased at 24 h and 48 h (108.5±15.97 24 h aged vs. 137.0±14.58 24 h aged+NMN; p<0.05; 77.25±11.53 48 h aged vs. 93.75±6.75 48 h aged+NMN; p = 0.1155; Fig 4B; n = 3). In addition, actin intensity was detected after oocytes were treated with cytochalasin B, and the signal was significantly reduced. Collectively, NMN rescued the decrease in actin signal intensity of aged porcine oocytes.

### 3.5 NMN reduced the abnormal chromosome morphology of aged porcine oocytes

**Fig 5:**
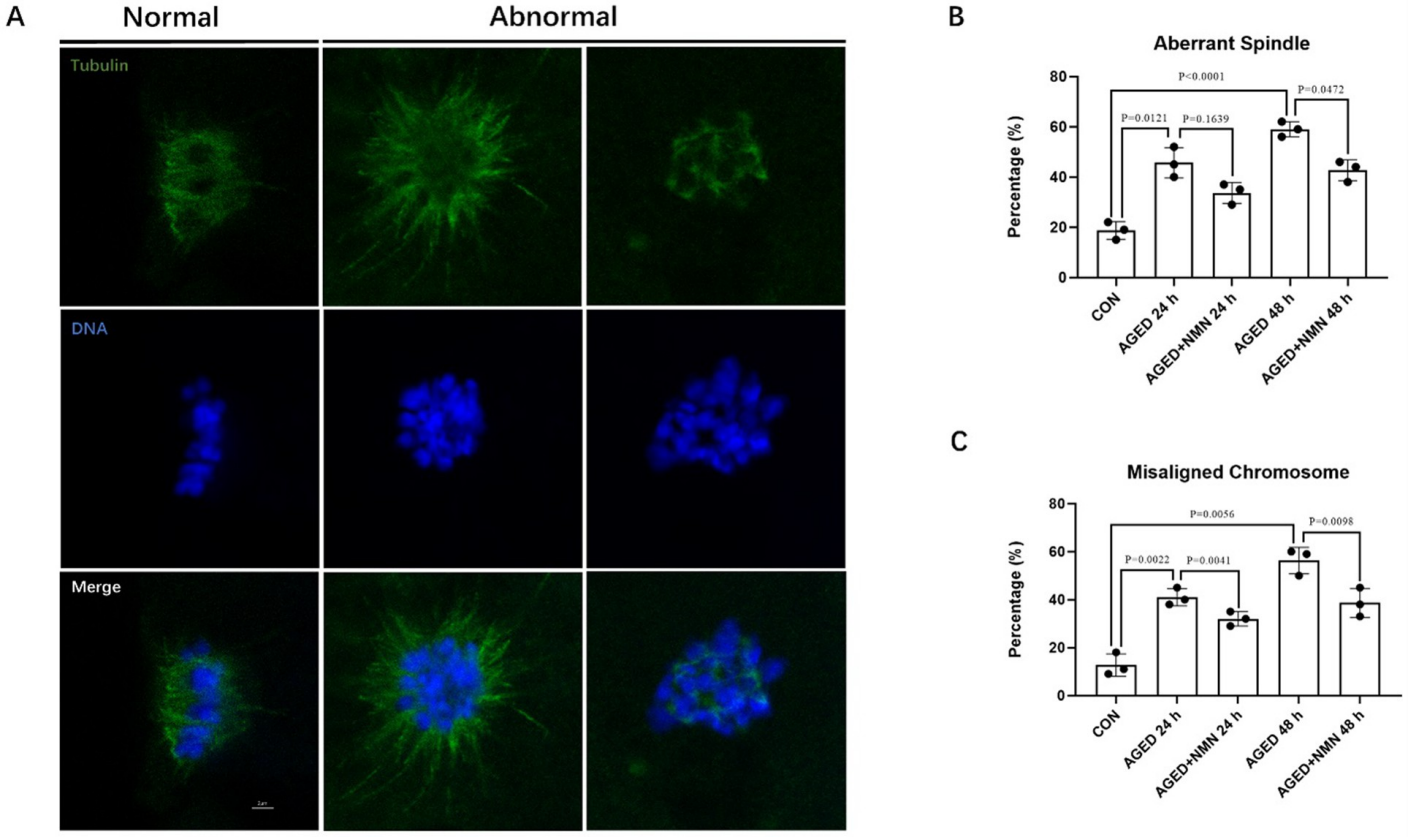
NMN maintained the chromosome morphology of aged porcine oocytes. (A) Representative confocal images of normal and abnormal spindles and DNA. (B, C) The ratio of abnormal spindle and misaligned chromosome at 0 h, AGED-24 h, AGED+NMN-24 h, AGED-48 h and AGED+NMN-48 h oocytes. *p < 0.05, **p < 0.01 indicated significant differences. Scale bars represented 2 μm.

In most cases, the quality of the oocyte is highly correlated with the proper organization of the cytoskeleton. The oocytes were stained with anti-α-tubulin FITC antibody and DApI respectively to observe the spindle morphology and chromosome arrangement. The normal spindle shape is symmetrically distributed on both sides of the chromosome, while the other forms are abnormal. The results of immunostaining showed that the proportion of abnormal spindle morphology and chromosome arrangement in senescent oocytes was much higher than that of fresh oocytes. However, NMN supplementation significantly reduced the proportion of abnormal spindle in 48 h aged oocytes (45.67±6.03% 24 h aged vs. 33.67±4.16% 24 h aged+NMN; p = 0.1639; 59.00±3.00% 48 h aged vs. 42.67±4.16% 48 h aged+NMN; Fig 5B; p<0.05; n = 3) and misaligned chromosome (41.00±3.61% 24 h aged vs. 32.00±3.00% 24 h aged+NMN; 56.33±5.51% 48 h aged vs. 38.67±6.03% 48 h aged+NMN; Fig 5C; p<0.01; n = 3) in aged porcine oocytes. Our results indicated that NMN alleviated the abnormality of spindle organization and chromosome arrangement of senescent oocytes.

### 3.6 NMN decreased the mRNA level of apoptosis-related genes of aged porcine oocytes

**Fig 6:**
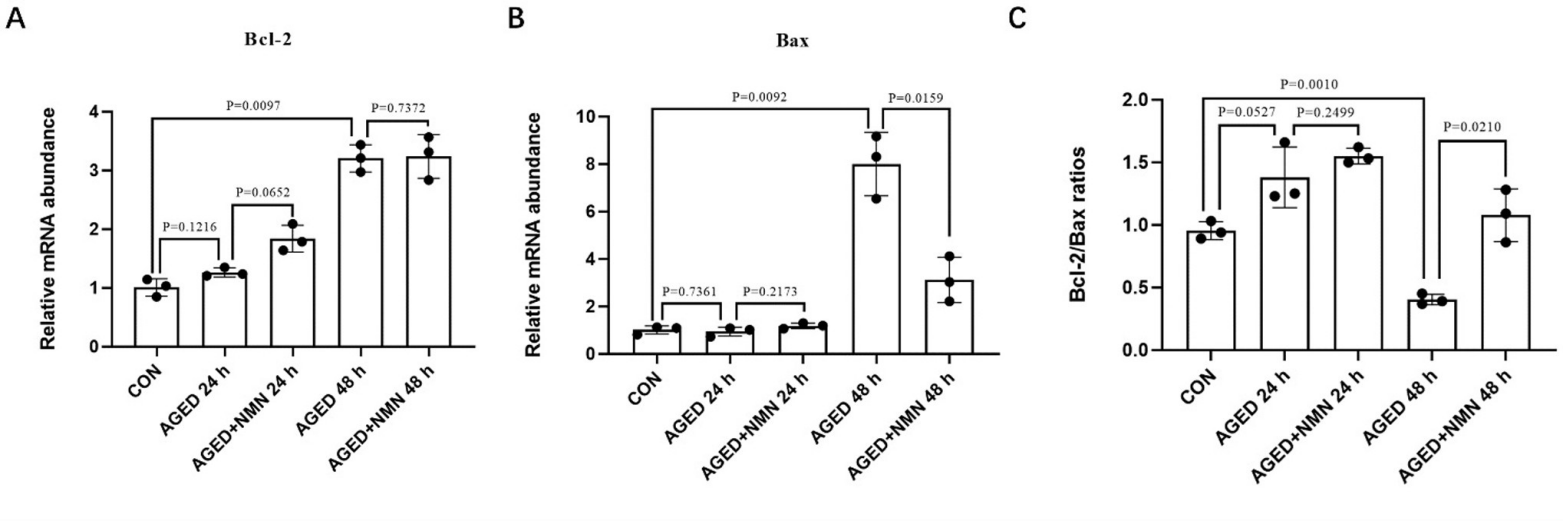
NMN decreased the mRNA level of apoptosis-related genes of aged porcine oocytes. (A) The expression of anti‐apoptosis genes (*Bcl2*) at 0 h, AGED-24 h, AGED+NMN-24 h, AGED-48 h and AGED+NMN-48 h oocytes. (B) The expression of pro‐apoptosis genes (*Bax*) at 0 h, AGED-24 h, AGED+NMN-24 h, AGED-48 h and AGED+NMN-48 h oocytes. (C) Effect of NMN on Bcl-2/ Bax ratio in aged oocytes. *p < 0.05, **p < 0.01 indicated significant differences.

Oxidative stress accelerates early oocyte apoptosis. The generation of oxidative stress will accelerate the early apoptosis of oocytes, and express the apoptosis-related genes *Bax* and *Bcl-2*. The results showed that the expression of anti-apoptotic gene *Bcl-2* in NMN treatment group showed no significant difference but increased trend at 24 h of aging (1.26±0.08% 24 h aged vs. 1.84±0.23% 24 h aged+NMN; Fig 6A; p = 0.065; n = 3). The expression of pro-apoptotic gene *Bax* in the NMN treatment group was significantly lower than that of the untreated group at 48 h, but there was no significant difference between the two groups at 24 h (0.94±0.19% 24 h aged vs. 1.18±0.11% 24 h aged+NMN; p = 0.217; 8.00±1.34% 48 h aged vs. 3.12±0.95% 48 h aged+NMN; Fig 6B; p<0.01; n = 3). Further analysis of Bcl-2/Bax ratio [24] showed that the Bcl-2/Bax ratio was significantly decreased when oocytes were aged for 48 h.

### 3.7 NMN improved the early embryonic development of aged oocytes

**Fig 7:**
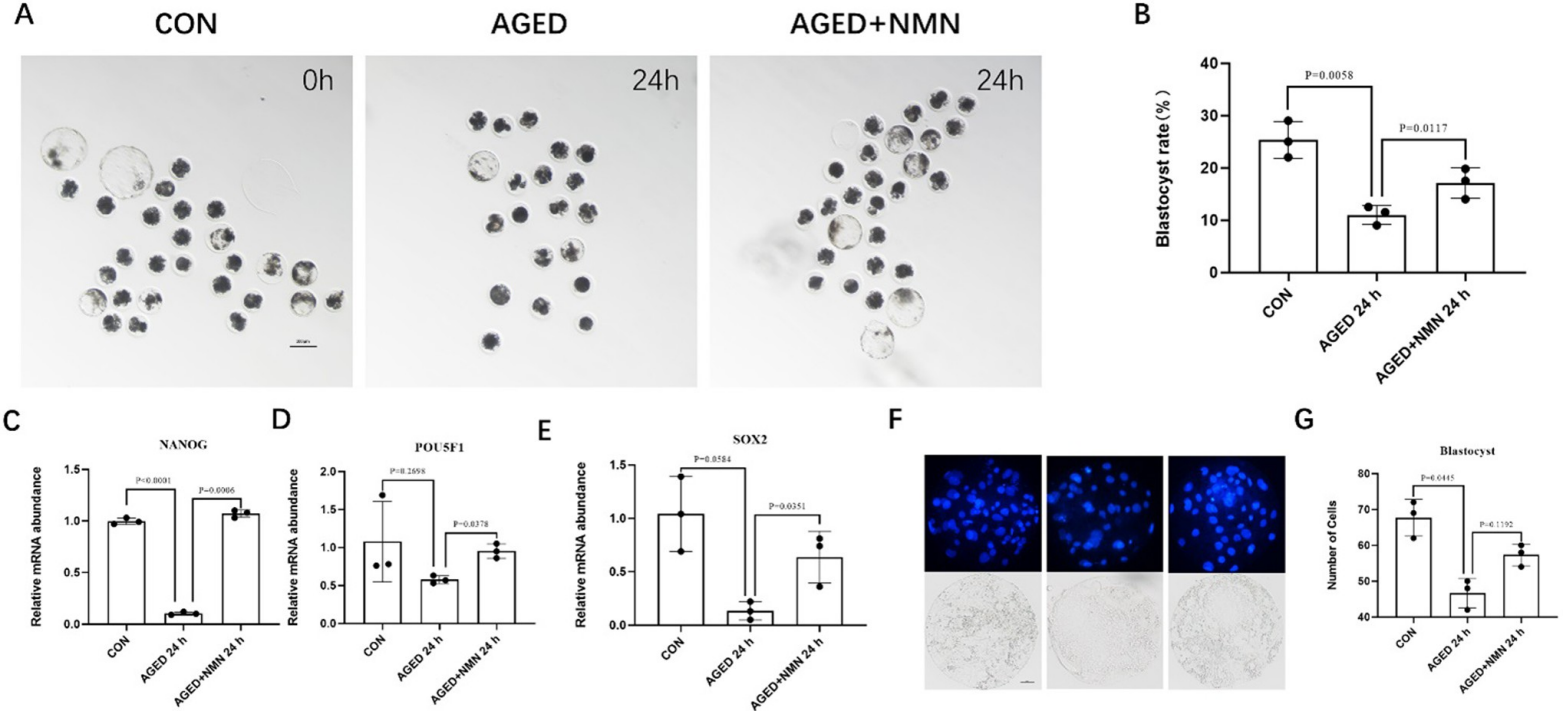
NMN improved the effects of aging on embryonic development. The D7 embryo morphologies (A) and blastocyst rate (B) at 0 h, AGED-24 h, AGED+NMN-24 h groups. Scale bars represented 100 μm. Expression of pluripotent genes *NANOG* (C) *OCT4* (D) and *SOX2* (E) at 0 h, AGED-24 h, AGED+NMN-24 h groups. *p < 0.05, **p < 0.01 indicated significant differences. (F) and (G) Statistics of the number of blastocysts at 0 h, AGED-24 h, AGED+NMN-24 h groups. Scale bars represented 50 μm.

Finally, in order to test whether NMN can improve the early embryonic development ability of aged oocytes, the oocytes supplemented by NMN were treated with parthenogenetic activation, and the subsequent embryo development was observed. The results showed that the rate of blastocysts was significantly reduced in oocytes aged for 24 h, and there was no blastocyst formation in oocytes aged for 48 h. After treated with NMN, the blastocyst rate in the NMN treatment group increased when compared with the aged group (25.33±3.51% control vs. 11.00±1.80% 24 h aged vs. 17.10±2.92% 24 h aged+NMN; Fig 7B; p<0.05; n = 3). Moreover, the expression levels of pluripotent genes *NANOG* (1.00±0.03% control vs. 0.10±0.01% 24 h aged vs. 1.07±0.03% 24 h aged+NMN; Fig 7C; p<0.001; n = 3), *OCT4* (1.08±0.53% control vs. 0.57±0.05% 24 h aged vs. 0.95±0.09% 24 h aged+NMN; Fig 7D; p<0.05; n = 3) and *SOX2* (1.04±0.35% control vs. 0.13±0.08% 24 h aged vs. 0.63±0.24% 24 h aged+NMN; Fig 7E; p<0.05; n = 3) are significantly reduced in oocytes aged for 24 h. However, compared with untreated aged group, NMN significantly increased the expression of pluripotent genes. In addition, there was no significant difference in the number of NMN treated blastocyst cells, but it still showed an upward trend (46.67±4.16 24 h aged vs. 57.33±3.01% 24 h aged+NMN; Fig 7G; p = 0.1192; n = 3).

## 4 Discussion

Oocyte senescence results in changes in the structure and function of mammalian oocytes, mainly including DNA damage, oxidative damage, abnormal chromosome arrangement and low fertility [25]. Here, we demonstrated NMN delayed oocyte senescence and promoted the development of subsequent embryos.

previously published microarray studies comparing old and young oocytes revealed altered expression of genes responsible for mitochondrial function, oxidative stress responses and chromosome alignment [26,27]. Another study showed that oocyte senescence accelerated ROS accumulation and mitochondrial damage, leading to an increased in the proportion of abnormal spindle filaments, while 200 mg/kg body weight/day NMN dose reduced ROS content, the proportion of abnormal chromosomes and improved mitochondrial function in aged mice oocyte [21]. While, in our study, we found that aging leads to ROS overexpression and mitochondrial dysfunction in porcine oocytes, consistent with previous reports in older mice, and 100 μmol/L NMN significantly reduces ROS levels in aging oocytes. Further study found that the expression levels of antioxidant genes *SOD1* and *Cat* were significantly increased in the NMN group. This explains that NMN can reduce ROS levels in aging oocytes by increasing the expression of antioxidant genes. In addition, NMN saves mitochondrial damage in aging oocytes and reduces abnormal spindle filaments and chromosome arrangement.

In addition to the age-related problems of decreased oocyte number and oocyte quality, preimplantation embryonic dysplasia also limited the number of euploid blastocysts used for transplantation in vitro [28]. It was found NMN supplementation improved blastocyst formation and blastocyst quality from aged mice oocytes [29]. However, it had no effect on the blastocyst formation of embryos derived from young mice oocytes, which was consistent with the conclusion of adding NMN in porcine embryo culture medium [30], indicating that NMN had no effect on cells with normal physiological functions. While, our study showed that prolonging the culture time of porcine oocytes significantly reduced the blastocyst formation, and NMN improved the development ability of porcine oocytes. Then we directly detected the expression of pluripotent genes *SOX2*, *OCT4* and *NANOG* in blastocysts. It was found that NMN increased blastocyst pluripotent gene expression in aged porcine oocytes. In addition, we measured the blastocyst number on day 7 and found that NMN increased the blastocyst number of senescent porcine oocytes. Therefore, we believed NMN improved the developmental ability of senescent oocytes, including blastocyst formation and quality after activation.

In summary, NMN reduces the ROS level of aged porcine oocytes and improves the mitochondrial function and the ability of embryo development. This study provides a new theoretical basis for clinical research to overcome the problem of human oocyte aged after ovulation.

## Supporting information

S1 File (XLSX)
